# Rescue analgesia with a transversus abdominis plane block alleviates moderate-to-severe pain and improves oxygenation after abdominal surgery: a randomized controlled trial

**DOI:** 10.3389/fpain.2024.1454665

**Published:** 2024-10-16

**Authors:** Jingxian He, Shuai Qin, Yuwen Wang, Qiuping Ye, Penglei Wang, Ye Zhang, Yun Wu

**Affiliations:** Department of Anesthesiology and Perioperative Medicine, The Second Affiliated Hospital of Anhui Medical University, Hefei, China

**Keywords:** rescue analgesia, transversus abdominis plane block, opioid, abdominal surgery, postoperative pain, oxygenation

## Abstract

**Background:**

Abdominal surgery is a common surgical procedure that is frequently associated with substantial postoperative pain. However, rescue analgesia using opioids is associated with several adverse effects. The transversus abdominis plane block (TAPB) has been demonstrated to be effective as part of multimodal analgesia. This study aims to evaluate the effects of rescue analgesia using the TAPB following abdominal surgery.

**Methods:**

Ninety patients undergoing abdominal surgery and reporting a numeric rating scale (NRS) score of cough pain ≥4 on the first postoperative day were randomized to receive either sufentanil or TAPB for rescue analgesia. Pain scores and arterial oxygen pressure (PaO_2_) were evaluated before and after the administration of rescue analgesia. Sleep quality and gastrointestinal function were assessed postoperatively. The primary outcome was the degree of pain relief on coughing 30 min after the administration of rescue analgesia.

**Results:**

Patients of both groups reported a significantly reduced NRS score on coughing 30 min after receiving rescue analgesia (*P*_paired_ < 0.001 for both groups). Notably, the degree of pain relief was significantly higher in the TAPB group than in the sufentanil group [median (interquartile range), −3 (−4 to −2) vs. −2 (−2 to −1), median difference = −1; 95% confidence interval, −2 to −1; *P* < 0.001]. Moreover, patients in the TAPB group experienced less pain than those in the sufentanil group during the following 24 h. When evaluated, PaO_2_ increased significantly after rescue analgesia was administered in the TAPB group (*P*_paired_ < 0.001); however, there were no significant intragroup differences in the sufentanil group (*P*_paired_ = 0.129). Patients receiving the TAPB experienced better quality of sleep than those receiving sufentanil (*P* = 0.008), while no statistical differences in gastrointestinal function were observed between the two groups.

**Conclusion:**

Rescue analgesia with the TAPB on the first postoperative day alleviated pain, enhanced oxygenation, and improved sleep quality in patients undergoing abdominal surgery; however, its effect on gastrointestinal function requires further research.

**Clinical Trial Registration:**

This study was registered in the Chinese Clinical Trial Registry (https://www.chictr.org.cn/showproj.html?proj=170983, ChiCTR2200060285) on 26 May 2022: Patients were recruited during the period between 30 May 2022 and 14 February 2023, and a follow-up of the last enrolled patient was completed on 16 March 2023.

## Introduction

In response to an increased focus on improving patient outcomes with surgical care, a growing body of clinical evidence, including postoperative pain control pathways, has recently been dedicated to enhanced recovery protocols (1). Abdominal surgery is one of the most frequent major surgeries performed worldwide. However, approximately 80% of patients suffer from mild to severe postoperative pain following open abdominal surgery or laparoscopic surgery (2, 3), especially after they leave the postanesthesia care unit (PACU). Poorly controlled postoperative pain is associated with increased morbidity and negatively affects quality of life (QoL) and functional recovery. In addition, postoperative pain in the abdominal region can cause shallow breathing and low cough effectiveness, leading to atelectasis and hypoxemia (4, 5). The administration of opioids to relieve pain is also associated with drug-related adverse effects such as postoperative nausea and vomiting (PONV), respiratory depression, and sleep disturbance (6, 7). Therefore, it is necessary to improve pain management to minimize these adverse effects and provide effective pain relief (8).

After the introduction of ultrasound in regional anesthetic practice, regional anesthetic procedures such as the transversus abdominis plane block (TAPB) and its derivative, the subcostal TAPB, are widely applied by practitioners in abdominal surgery because of their favorable analgesic efficacy (9). However, there is a lack of research on the use of the TAPB as a rescue analgesic method after surgery. Therefore, in this study, we designed a randomized controlled trial (RCT) to compare the efficacy of the TAPB with that of opioids as rescue analgesia delivered on the first postoperative day in patients who had undergone abdominal surgery. Our primary focus was to analyze the level of pain relief experienced by patients during coughing 30 min after rescue analgesia was administered. We hypothesized that pain scores would be further reduced in patients receiving the TAPB.

## Methods

### Study design

This randomized controlled trial was conducted between May 2022 and February 2023. Approval was given by the Ethics Committee of the Second Affiliated Hospital of Anhui Medical University (approval no.: YX2022-49), and it was prospectively registered in the Chinese Clinical Trial Registry (https://www.chictr.org.cn/showproj.html?proj=170983, registration number: ChiCTR2200060285) on 26 May 2022. The study was performed in accordance with the criteria of the Consolidated Standards of Reporting Trials (CONSORT) (10) and with those of the Declaration of Helsinki. Written informed consent was obtained from all patients before their participation in the study.

Patients who underwent abdominal surgery and reported a numeric rating scale (NRS) score of cough pain ≥4 on the first postoperative day were enrolled in this study. The inclusion criteria were patients aged ≥18 years with physical status grades I–III, based on the American Society of Anesthesiologists (ASA). The exclusion criteria were a body mass index (BMI) >35 kg/m^2^, daily use of opioid analgesics, presence of coagulopathy, known allergy to any of the study drugs, infection at the injection area, mental or neurological disorders, or severe cardiovascular system disease.

### Randomization and blinding

The enrolled patients were randomly assigned to either the opioid or the TAPB group using SPSS (version 26.0; IBM, Armonk, NY, USA) at a 1:1 ratio. A randomization list was prepared in consecutively numbered, sealed, and opaque envelopes by an assistant who was not involved in the study. A consultant anesthesiologist who was not involved in data collection or analysis opened the envelopes to reveal the group allocation shortly before rescue analgesia was administered. This anesthesiologist had previously performed over 200 TAPB procedures and provided rescue analgesia (opioid or TAPB) in accordance with the randomization list. In addition, this anesthesiologist had also performed preoperative TAPB procedures for all patients. Thereafter, the nursing staff, attending anesthesiologists, and outcome assessors were blinded to the patient group allocation and did not have access to group designation until the data analysis was complete.

### General anesthesia technique

**Figure 1:**
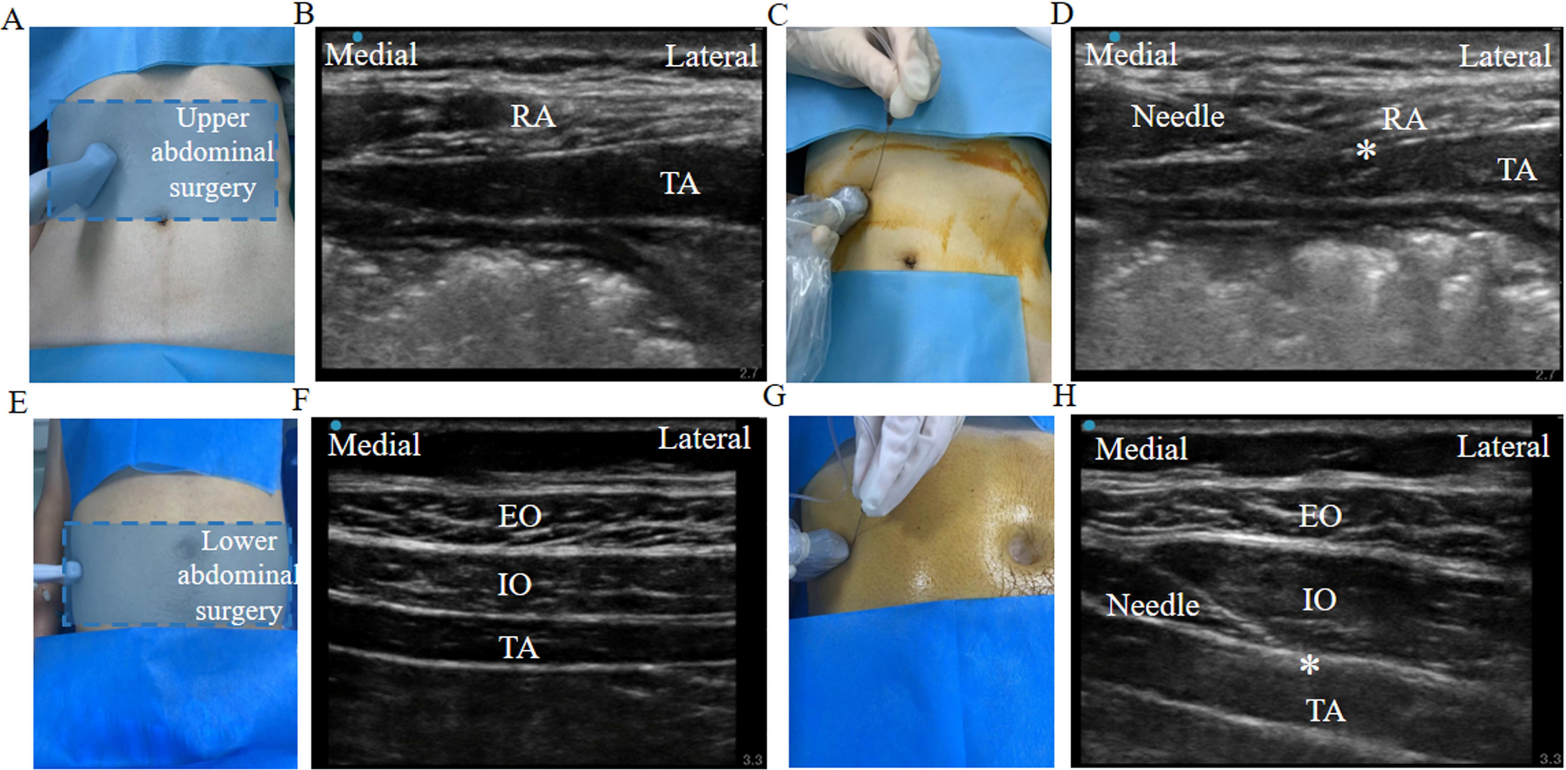
A schematic diagram of the TAPB. **(A–D)** The ultrasound probe position, the needle puncture site, and a sonographic image of the subcostal TAPB for upper abdominal surgery. The asterisk indicates the needle target. **(E–H)** The ultrasound probe position, the needle puncture site, and a sonographic image of the lateral TAPB for lower abdominal surgery. TAPB, transversus abdominis plane block; RA, rectus abdominis muscle; TA, transversus abdominis muscle; EO, external oblique muscle; IO, internal oblique muscle.

After the patients arrived in the operating room, intravenous access was established with an 18-gauge intravenous cannula, and infusion of lactated Ringer's solution was initiated. Standard monitoring procedures such as blood pressure measurement, electrocardiogram, and pulse oximetry were performed. All patients received ultrasound-guided peripheral nerve blocks before anesthesia. Our standard practice during the study period was to perform the TAPB procedure using the subcostal and lateral approach for abdominal surgery (11). The TAPB procedure via the subcostal approach was performed in patients scheduled for upper abdominal surgery, including gastrectomies and hepatobiliary, splenic, and pancreatic surgeries. During the procedure, a linear ultrasound transducer (13–6 MHz) covered with sterile transparent plastic was positioned at the midline and moved laterally along the subcostal margin to identify the plane between the transversus abdominis and the rectus abdominis muscles (Figures 1A–D). After negative aspiration, 20 ml of 0.25% ropivacaine was injected between the plane of these two muscles at the point of the lateral end of the rectus abdominis muscle (Figures 1A–D). The procedure was then repeated on the contralateral side. The TAPB procedure using a lateral approach was performed in patients scheduled for colorectal surgery. The probe was positioned in the midaxillary line between the iliac crest and the costal margin. The needle was inserted in-plane and advanced anterior-to-posterior under continuous visualization until the tip was visualized between the internal oblique and the transversus abdominis muscles (Figures 1E–H). A quantity of 20 ml of 0.25% ropivacaine was then injected between the plane of these two muscles and the procedure was repeated on the contralateral side.

After the nerve block procedure, the patients were administered general anesthesia, which was induced with midazolam (0.05 mg/kg), sufentanil (0.4 μg/kg), etomidate (0.3 mg/kg), and cisatracurium (0.2 mg/kg). Intravenous dexamethasone (8–10 mg) was administered to prevent PONV. Maintenance of anesthesia was achieved by continuous infusion with propofol (4–6 mg/kg/h), remifentanil (0.15–0.25 μg/kg/min), and inhalation of sevoflurane (0.6%–1.0%). Muscle relaxation was achieved using cisatracurium (weight-adjusted dosing by the attending anesthesiologist). The depth of the anesthesia was adjusted to maintain a bispectral index target range of 40–60. The anesthesiologists administered intravenous sufentanil (5–10 μg) when the patients’ heart rate or blood pressure increased by >20% from basal measurements.

All patients were transferred to the anesthesia intensive care unit (AICU) for recovery after extubation, and a standardized postoperative analgesia protocol that included sufentanil postoperative-controlled intravenous analgesia (PCIA) and intravenous rescue analgesics (pentazocine and flurbiprofen) was initiated. Sufentanil at a concentration of 1.5 μg/ml was included in our PCIA protocol (total volume 100 ml). PCIAs were set to a basal infusion of 2 ml/h and 1.2 μg bolus doses with a 15 min lock time. All patients were provided a detailed explanation on how to use the PCIA pump and educated to report pain intensity using a 10-point NRS, which ranged from “0” (meaning no pain) to “10” (meaning worst pain imaginable). The PCIA button was pressed when the NRS score at rest was ≥4 or patients verbalized the need for pain relief. If three boluses of sufentanil did not alleviate pain, pentazocine 30 mg was administered intravenously as rescue analgesia. Another bolus of intravenous flurbiprofen 50 mg was administered for further analgesia if pain persisted for 30 min after pentazocine administration.

### Rescue analgesia

On the morning of the first postoperative day, the patients reported their pain NRS scores. When the NRS score on coughing was ≥4, the consultant anesthesiologist who performed the preoperative TAPB reviewed and provided rescue analgesia in accordance with the randomization list. The patients were placed in a supine position and oxygen was provided at 2 L/min through a nasal cannula. In the sufentanil group (CON group), boluses of sufentanil (0.1 μg/kg) were injected intravenously. In the TAPB group, an ultrasound-guided TAPB or subcostal TAPB was performed depending on the surgical procedure. In the same way as the preoperative TAPB, patients who had undergone gastrectomy, or hepatobiliary, splenic, or pancreatic surgery received the TAPB via the subcostal approach. A total of 20 ml of 0.25% ropivacaine was injected on each side of the plane between the transversus abdominis and the rectus abdominis muscles. Patients who had undergone colorectal surgery received the TAPB via the lateral approach. Local anesthetic was injected on each side of the plane between the internal oblique and the transversus abdominis muscles (11). Pain NRS scores were recorded after the administration of rescue analgesia. Arterial oxygen pressure (PaO_2_) and arterial carbon dioxide pressure (PaCO_2_) were measured using arterial blood gas samples before and 30 min after rescue analgesia was administered.

### Postoperative management

Once the AICU discharge criteria were met, patients were transferred to the ward and PCIA was used as needed. Non-steroidal anti-inflammatory drugs were provided to the patients as needed for pain relief, as reviewed and decided by the attending surgeons. The patients were interviewed 24 h after receiving rescue analgesia to obtain pain NRS scores. Perceived nighttime sleep quality on the first postoperative day was evaluated using the Richards–Campbell Sleep Questionnaire (RCSQ) (12). The RCSQ responses were recorded on a 100-mm visual analog scale, with higher scores representing better sleep. Gastrointestinal function was evaluated at 72 h after surgery. Based on the clinical presentation of the patient, the scoring system assigns 0–2 points to each of the five components: intake, feeling nauseated, emesis, physical examination, and duration of symptoms (I-FEED) (13). Here, the patients were categorized as follows: normal (0–2), postoperative gastrointestinal function intolerance (3–5) (POGI), and postoperative gastrointestinal dysfunction (≥6) (POGD). PONV was assessed using the following scores: 0 = no PONV, 1 = mild nausea, 2 = moderate nausea, 3 = severe nausea/mild vomiting, and 4 = continuous vomiting. Rescue droperidol was administered for PONV. At 1 month after surgery, the patients’ QoL was assessed using the Spitzer QoL index, which covers five domains: involvement in own occupation (activity), activities of daily living (daily living), perception of own health (health), support of family and friends (support), and outlook on life (outlook) (14). A total score, ranging from 0 to 10, is the sum of item scores, and a higher score represents a better QoL.

### Outcomes

The primary outcome was the degree of pain relief on coughing 30 min after the administration of rescue analgesia. The secondary outcomes were NRS pain scores at rest and on coughing, evaluated at 0.5, 3, 6, and 24 h after the patients received rescue analgesia; PaO_2_ and PaCO_2_ measured at 30 min after rescue analgesia was given; nighttime sleep quality on the first postoperative day; postoperative sufentanil consumption; frequency of PCIA; PONV score; time to first postoperative flatus and ambulation; gastrointestinal function; postoperative complications; and QoL.

### Sample size calculation

The sample size was calculated based on the primary outcome by using PASS (version 15.0; PASS, NCSS, USA) for Windows. According to our pilot study (*n* = 12 in each group), the mean ± standard deviations (SDs) of the pain relief scores were −3.0 ± 1.3 for the TAPB group and −2.0 ± 1.3 for the control group, and two independent means were compared. Considering a power of 0.90, an alpha error of 0.05, and assuming a dropout rate of 20%, we recruited 90 patients into the study.

### Statistical analysis

We used SPSS (version 26.0; IBM, Armonk, NY, USA) for statistical analysis. A Kolmogorov–Smirnov test and a visual inspection of histograms were used to test the assumption of normality. Continuous variables are expressed as mean (SD) or median (interquartile range). Student's *t*-test was used to compare parametric variables with a normal distribution between the two groups. Comparisons for the changes in NRS pain, PaO_2_, and PaCO_2_ scores were analyzed using the Mann–Whitney *U* test and by calculating the Hodge–Lehman median difference with a constructed 95% confidence interval (CI). Categorical data are described as numbers (%), and a chi-square or Fisher's exact test was used for determining intergroup differences as appropriate. A paired Wilcoxon test was used for binary comparisons of variables within the groups. Repeated measurements of postoperative pain scores were analyzed using a linear mixed model (15, 16) to evaluate the association between the NRS pain score over time and the rescue analgesia method. Intervention, time, and the interaction between time and intervention were set as the fixed effects. Time was included as a repeated effect and NRS pain scores were included as dependent variables. Assessments were two-sided, and *P*-values <0.05 were considered statistically significant.

## Results

**Figure 2:**
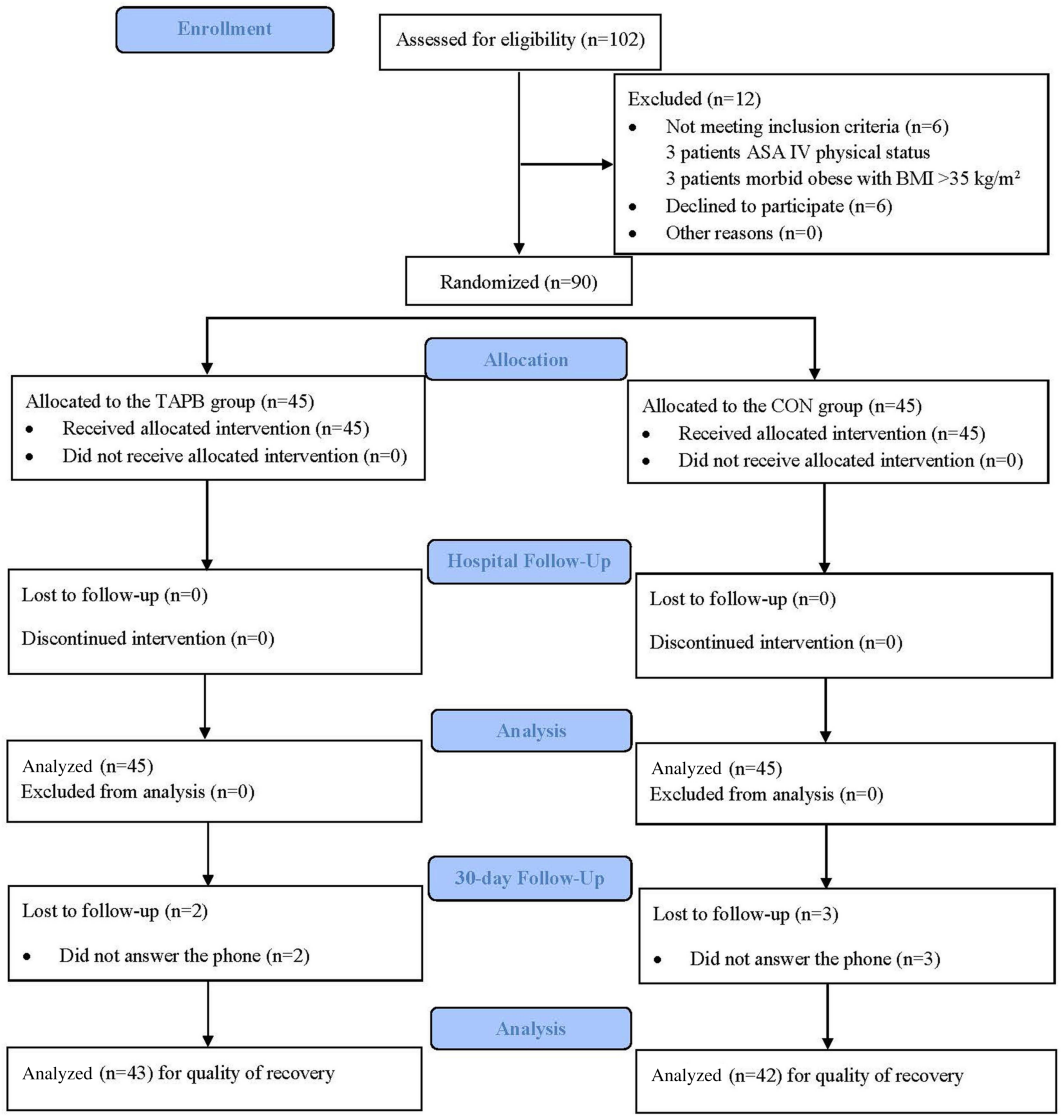
A consolidated standards of reporting trials flow diagram of participants through each stage of the randomized trial. ASA, American Society of Anesthesiologists; BMI, body mass index; TAPB, transversus abdominis plane block; CON, control.

Figure 2 presents the CONSORT flow diagram for this trial. Patients were recruited during the period between 30 May 2022 and 14 February 2023, and a follow-up of the last enrolled patient was completed on 16 March 2023. A total of 102 patients were evaluated for enrollment in the study. Six patients did not meet the inclusion criteria (three patients had an ASA IV physical status and three patients were morbidly obese with a BMI > 35 kg/m^2^), and six patients declined to participate in the study. Thus, 90 patients were enrolled and randomized. However, five patients (two patients in the TAPB group and three patients in the CON group) were lost to the 30-day follow-up; thus, 85 patients were analyzed for the outcome of the 30-day follow-up.

The general and surgical characteristics of the patients are summarized in Table 1. We found no significant differences between the two groups in terms of age, sex distribution, BMI, ASA physical status, preoperative comorbidities, type of surgery, proportion of open abdominal surgery and laparoscopic surgery, or duration of surgery (Table 1). The time interval between preoperative TAPB and rescue analgesia on the first postoperative day was comparable between the two groups. Prior to the administration of rescue analgesia on the first postoperative day, there were no significant differences between the groups in the consumption of intraoperative analgesics, PCIA (sufentanil), pentazocine, or flurbiprofen used in the AICU (Table 2).

**Table 1:** Patient demographic characteristics and surgical parameters.

	TAPB group (*n* = 45)	CON group (*n* = 45)	Standardized difference	*P*-value
Age, years	63.6 (10.5)	64.4 (13.9)	0.07	0.745
Sex, male, *n* (%)	27 (60.0)	25 (55.6)	0.09	0.670
Body mass index (kg/m^2^)	23.2 (3.1)	22.1 (3.3)	0.34	0.109
ASA physical status, *n* (%)			0.12	0.561
II	39 (86.7)	37 (82.2)		
III	6 (13.3)	8 (17.8)		
Type of surgery, *n* (%)				0.338
Gastric	17 (37.8)	12 (26.7)	0.24	
Colorectal	14 (31.1)	17 (37.8)	0.14	
Hepatic	9 (20.0)	10 (22.2)	0.05	
Biliary	1 (2.2)	4 (8.9)	0.30	
Splenic	4 (8.9)	1 (2.2)	0.30	
Pancreatic	0 (0.0)	1 (2.2)	0.21	
Open abdominal surgery	24 (53.3)	25 (55.6)	0.05	0.832
Duration of surgery (min)	245 (91)	244 (113)	0.01	0.976
Preoperative comorbidities				
Hypertension	13 (28.9)	12 (26.7)	0.05	0.814
Diabetes mellitus	7 (15.6)	3 (6.7)	0.29	0.180
Coronary artery disease	2 (4.4)	3 (6.7)	0.10	>0.999
Cerebrovascular disease	3 (6.7)	0 (0.0)	0.38	0.242
Asthma	1 (2.2)	1 (2.2)	0	>0.999
Anemia	1 (2.2)	1 (2.2)	0	>0.999
Arrhythmia	1 (2.2)	1 (2.2)	0	>0.999

TAPB, transversus abdominis plane block; CON, control; ASA, American Society of Anesthesiologists.

Data are presented as mean (standard deviation) or *n* (%).

**Table 2:** Analgesia parameters before intervention.

	TAPB group (*n* = 45)	CON group (*n* = 45)	*P*-value
Time interval of the TAPB/opioid (h)	22.0 (2.7)	21.2 (3.5)	0.249
Consumption of analgesics before intervention			
Intraoperative sufentanil consumption (μg)	38.6 (9.3)	35.3 (9.1)	0.099
Intraoperative remifentanil consumption (mg)	3.8 (1.5)	4.1 (1.6)	0.277
Sufentanil consumption of PCIA (μg)	31.4 (5.7)	33.3 (5.6)	0.104
Pentazocine (mg)	0 (0, 30)	0 (0, 30)	0.832
Flurbiprofen (mg)	0, (0, 0)	0, (0, 0)	0.505

TAPB, transversus abdominis plane block; CON, control; IV, intravenously.

Data are presented as mean (standard deviation) and median (interquartile range).

A score of *P* < 0.05 indicates statistically significant differences between the two groups.

**Figure 3:**
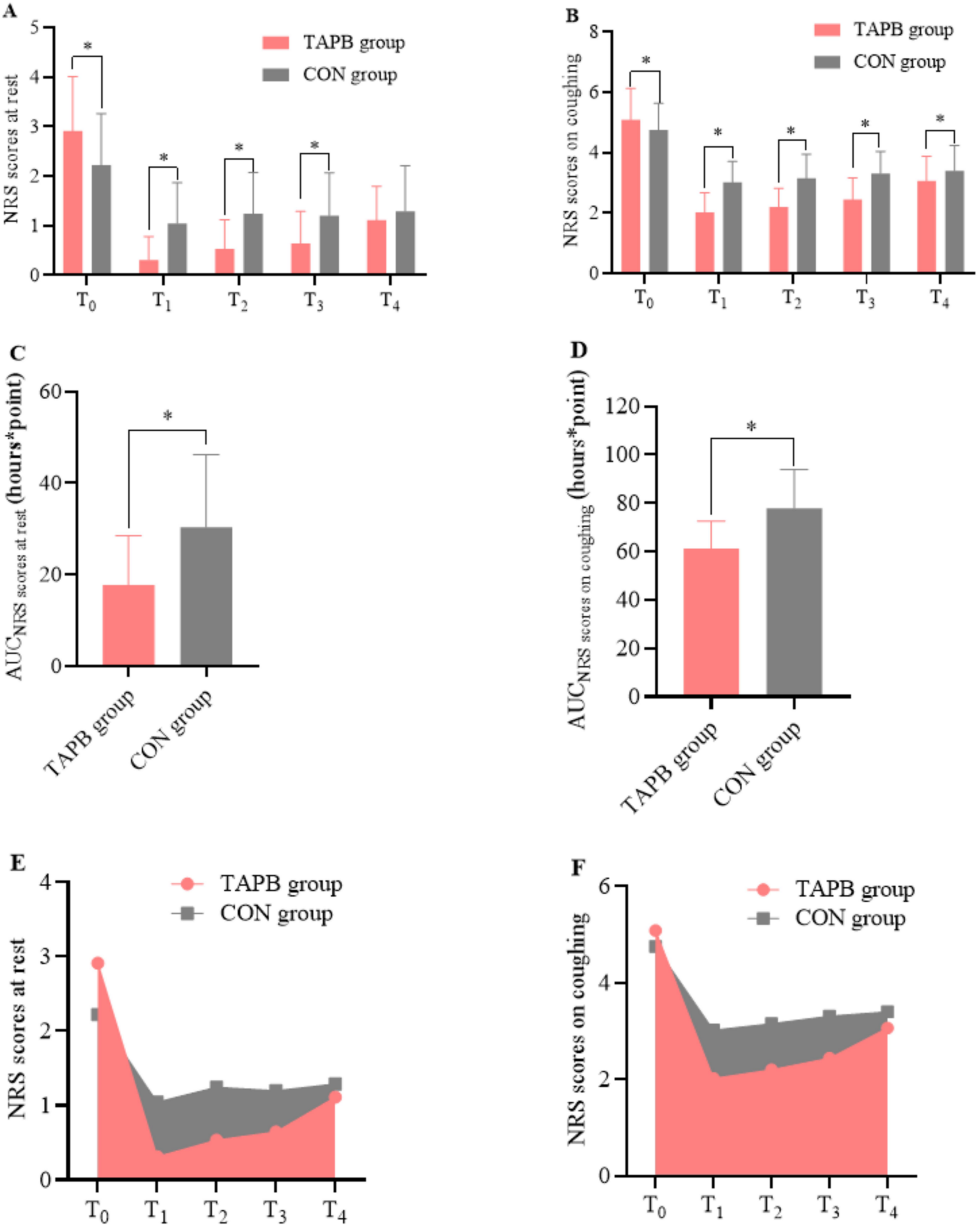
NRS pain scores and the AUC of scores in the studied groups. Data are expressed as mean (standard deviation). Comparisons between the patient groups were made using linear mixed-model analyses. Patients in the TAPB group had a higher NRS score at rest and on coughing than those in the control group before receiving rescue analgesia. After the administration of rescue analgesia, the TAPB group exhibited significantly reduced pain scores at rest during 6 h **(A)** and on coughing during 24 h following the administration **(B)** The AUC of the NRS score at rest and on coughing was significantly reduced in the TAPB group **(C–F)**. *P < 0.05 indicates statistically significant differences compared with the control group. *P*-values are corrected using Bonferroni correction. TAPB, transversus abdominis plane block; CON, control; NRS, numeric rating scale; AUC, area under the curve. T_0_, before rescue analgesia; T_1_, 30 min after rescue analgesia; T_2_, 3 h after rescue analgesia; T_3_, 6 h after rescue analgesia; T_4_, 24 h after rescue analgesia.

Patients in both groups reported significantly reduced NRS scores 30 min after receiving rescue analgesia (*P*_paired_ < 0.001 for both groups). Notably, the degree of pain relief at rest and on coughing were significantly higher in the TAPB group than in the CON group [NRSrest: −2 (−3 to −2) vs. −1 (−2 to −1), mean difference = −1, 95% CI, −2 to −1, *P* < 0.001; NRS coughing: −3 (−4 to −2) vs. −2 (−2 to −1), mean difference = −1, 95% CI, −2 to −1, *P* < 0.001]. The PaO_2_ increased significantly after rescue analgesia was administered in the TAPB group (*P*_paired_ < 0.001); however, there were no significant intragroup differences in the sufentanil group (*P*_paired_ = 0.129). Meanwhile, the TAPB group exhibited a greater elevation of PaO_2_ levels than the CON group [9 (4 to 20) vs. 3 (−2 to 10), mean difference = 7, 95% CI, 3–11, *P* < 0.001]. Patients receiving the TAPB experienced a significant decrease in PaCO_2_ after receiving rescue analgesia, while there were no significant differences in PaCO_2_ before and after receiving rescue analgesia in the CON group (Table 3). We also found that the TAPB group had significantly lower NRS scores than the CON group during the following 24 h (Figures 3A,B). Moreover, pain area under the curve at rest and on coughing in the TAPB group were significantly reduced compared with those in the CON group (Figures 3C–F).

**Table 3:** NRS pain score and PaO_2_ and PaCO_2_ levels before and 30 min after rescue analgesia administration.

	TAPB group (*n* = 45)	CON group (*n* = 45)	Mean (median) difference (95% CI)	*P*-value
NRS rest				
Pre	2.9 (1.1)	2.2 (1.0)	0.7 (0.2 to 1.1)	0.003
Post	0.3 (0.5)	1.0 (0.8)	−0.7 (−1.0 to −0.5)	<0.001
ΔNRS rest	−2 (−3 to −2)	−1 (−2 to −1)	−1.0 (−2.0 to −1.0)	<0.001
*P*_paired_	<0.001	<0.001		
NRS coughing				
Pre	5.1 (1.0)	4.8 (0.9)	0.3 (−0.1 to 0.7)	0.105
Post	2.0 (0.7)	3.0 (0.7)	−1.0 (−1.3 to −0.7)	<0.001
ΔNRS coughing	−3 (−4 to −2)	−2 (−2 to −1)	−1.0 (−2.0 to −1.0)	<0.001
*P*_paired_	<0.001	<0.001		
PaO_2_				
Pre	106.6 (23.9)	111.6 (26.8)	−4.9 (−15.6 to 5.7)	0.359
Post	119.6 (25.4)	114.2 (25.1)	5.4 (−5.2 to 16.0)	0.313
ΔPaO_2_	9 (4, 20)	3 (−2, 10)	7.0 (3.0 to 11.0)	<0.001
*P*_paired_	<0.001	0.129		
PaCO_2_				
Pre	40.0 (5.1)	38.6 (4.6)	1.5 (−0.6 to 3.5)	0.153
Post	38.5 (3.9)	38.0 (3.8)	0.4 (−1.2 to 2.0)	0.603
ΔPaCO_2_	−1 (−4 to 0)	−1 (−2 to 2)	−1.0 (−2.0 to 0.0)	0.136
*P*_paired_	<0.001	0.239		

NRS, numeric rating scale; TAPB, transversus abdominis plane block; CON, control.

Data are presented as mean (standard deviation) and median (interquartile range).

A score of *P* < 0.05 indicates statistically significant differences between the two groups. A score of *P*_paired_ < 0.05 indicates statistically significant differences compared with prerescue analgesia values.

Patients who received the TAPB experienced a significantly higher quality of sleep than those who received opioids [36.4 (6.8) vs. 33.0 (5.2), *P* = 0.008]. The PONV score was also significantly lower in the TAPB group than that in the CON group [0 (0–1) vs. 1 (1–1), *P* = 0.013]. However, no statistical differences were observed between the two groups in any of the following secondary outcomes: frequency of PCIA, postoperative sufentanil and flurbiprofen consumption, time to first postoperative flatus and ambulation, gastrointestinal function, postoperative complications, or QoL (Table 4).

**Table 4:** Postoperative parameters.

	TAPB group (*n* = 45)	CON group (*n* = 45)	*P*-value
Frequency of PCIA	0 (0, 1)	0 (0, 2)	0.181
Postoperative sufentanil consumption (μg)	72.9 (1.5)	73.5 (2.4)	0.145
Postoperative flurbiprofen consumption (mg)	0 (0, 50)	0 (0, 50)	0.779
Time to first postoperative flatus (h)	61.4 (28.8)	67.8 (34.3)	0.342
Time to first ambulation (h)	62.7 (33.1)	76.9 (45.7)	0.095
Sleep quality	36.4 (6.8)	33.0 (5.2)	0.008
PONV score	0 (0, 1)	1 (1, 1)	0.013
I-FEED, *n* (%)			0.948
Normal	16 (35.6)	14 (31.1)	
POGI	25 (55.6)	27 (60.0)	
POGD	4 (8.9)	4 (8.9)	
Postoperative complications, *n* (%)			
Delirium	2 (4.4)	0 (0.0)	0.494
Atrial fibrillation	0 (0.0)	1 (2.2)	>0.999
Pleural effusion	0 (0.0)	1 (2.2)	>0.999
Lung infection	0 (0.0)	1 (2.2)	>0.999
QoL at 30 days after surgery^a^	9.0 (0.8)	8.6 (1.6)	0.112

TAPB, transversus abdominis plane block; CON, control; PCIA, patient-controlled intravenous analgesia; PONV, postoperative nausea and vomiting; I-FEED, intake, feeling nauseated, emesis, physical examination, and duration of symptoms; POGI, postoperative gastrointestinal function intolerance; POGD, postoperative gastrointestinal dysfunction; QoL, quality of life.

Data are presented as mean (standard deviation), median (interquartile range), or *n* (%).

^a^ *n* = 43 in the TAPB group, *n* = 42 in the CON group.

## Discussion

This prospective randomized study demonstrated that rescue analgesia with the TAPB on the first postoperative day provided significantly greater pain relief for patients undergoing abdominal surgery than opioids. Moreover, the TAPB helped improve oxygenation and sleep quality and reduced the degree of PONV. However, we observed no intergroup differences in postoperative opioid consumption, gastrointestinal function, or QoL.

Significant abdominal wall pain is common after abdominal surgery and necessitates comprehensive multimodal pain management strategies (17). Since they were first described by Rafi in 2001 (18), TAPBs have become one of the most commonly performed truncal blocks to achieve pain relief and opioid-sparing analgesia in abdominal surgeries. The TAPB procedure can be performed using several different approaches, including the subcostal approach, which covers the 6th to 9th thoracic dermatomes and provides analgesia to the upper abdomen, and the lateral approach, which covers the 10th to 12th thoracic dermatomes and provides lower abdominal wall analgesia (19). As a standard practice at our institution, we performed a preincisional TAPB using the subcostal or the lateral approach according to the abdominal incision to provide preemptive analgesia for patients undergoing abdominal surgery (20).

However, single-shot TAPB is of limited duration, which makes it unsuitable for the management of significant and prolonged postoperative pain (21). Although patients often receive PCIA for postoperative analgesia, the efficacy of PCIA is variable (22, 23). It has been reported that 30% of patients presented a prolonged moderate to severe pain at mobilization 24 h after abdominal surgery (24). According to the medical records in our institution, approximately 34% of patients continue to rate their pain as worse than 4/10 during the early postoperative period. Thus, rescue analgesia with multimodal methods is often needed. On the day of the surgery, the patients received intravenous pentazocine and flurbiprofen for rescue analgesia. Considering the side effects of frequent systemic administration for analgesia, we assessed the efficacy of the TAPB procedure as a rescue analgesia method for pain relief on the first postoperative day after abdominal surgery. We found that both approaches of the TAPB provided satisfactory pain relief in patients reporting an NRS score of cough pain ≥4. Furthermore, patients receiving the TAPB experienced a significantly greater pain relief and a lesser degree of PONV than those receiving opioids, demonstrating that the TAPB is a prospective method for rescue analgesia after abdominal surgery.

Postoperative pain can induce various pulmonary complications by decreasing ciliary function, limiting respiratory effort, and lowering cough effectiveness, predisposing patients to atelectasis and hypoventilation (25, 26). Opioids are the most potent analgesic in the clinic. However, opioids may depress the central neural drive to the respiratory muscles and decrease the sensitivity to carbon dioxide, leading to respiratory depression and cough inhibition (27, 28). Previous studies have demonstrated that peripheral nerve blocks significantly improve oxygenation and pulmonary function in patients undergoing thoracic and open heart surgeries (27, 29). Consistently, our study revealed a significant difference between groups regarding respiratory functions, with a higher degree of PaO_2_ increase in the TAPB group than in the control group. This may be attributed to the greater pain relief in patients receiving the TAPB procedure, leading to an improvement in coughing and deep breathing, while avoiding respiratory depression produced by systemic opioids (30, 31).

Postoperative pain leads to poor sleep, while sleep disturbance reciprocally induces hyperalgesia and exacerbates clinical pain (32). However, pain control with opioid medications can also complicate the management of sleep disorders such as insomnia and sleep-disordered breathing (33, 34). It has been demonstrated that sleep disturbance was associated with opioid use in a large cohort of chronic non-cancer pain patients receiving long-term opioid therapy (35). While in a randomized control study, opioid-free analgesia with the TAPB and esketamine exhibited advantages of lower incidence of PONV and higher quality of sleep in patients undergoing gynecological laparoscopic surgery. Consistently, the results of our study showed that rescue analgesia with the TAPB also improved sleep quality. These findings further suggest that the TAPB may be a favorable alternative to opioids for perioperative pain management.

Patients in the TAPB group showed a slight reduction in cumulative opioid consumption in the first 24 h after receiving rescue analgesia, but this was not significant; however, they reported a significant decrease in pain scores. We attributed the non-significant opioid reduction to mild pain intensity (NRS score <4) after rescue analgesia. Furthermore, patients usually consider suffering from pain as a natural phenomenon and may be unaware of the importance of effective pain management after surgery. Meanwhile, given the fear of dependence on opioids and the related adverse reactions, patients are more likely to tolerate pain and seldom press the PCIA button (36–38). Since perioperative cumulative opioid consumption was comparable between the two groups, we also did not find any promising results for the first postoperative flatus or ambulation and gastrointestinal function or QoL at 1 month after surgery. Further research is required to evaluate the efficacy of the TAPB as a rescue analgesic for opioid consumption and postoperative recovery.

Since the TAPB is a kind of surgical procedure, it should be avoided when there is reliable medication to relieve pain. Considering the higher risk of a patient developing severe complications during the early postoperative period, patients undergoing major abdominal surgery in our institution were transferred to the AICU for overnight monitoring and treatment. However, in most cases, patients were transferred to the ward from the PACU. It is often difficult to perform invasive procedures in these environments because of the constraints of space and logistics and the challenges involved in infection control. Meanwhile, the limited duration of single-shot TAPB may necessitate repeated procedures. We evaluated the performance of nerve blocks only on the morning of the postoperative day, and challenges in administering the TAPB during night shifts should be considered. The use of continuous infusions via indwelling catheters after surgery could potentially extend the duration of analgesia and improve pain management overnight, without causing delays in the administration of rescue analgesia due to the time needed for patient call (21). However, the added risk of infection, as well as requiring specialized infusion pumps and monitoring, should not be ignored (39). Future research in this area is required to evaluate the efficacy of the TAPB procedure in different delivery methods.

Our study had a few limitations. First, awake patients might distinguish the method of intervention they received. To minimize biases, all outcome assessors were blinded to patient group allocation. Second, the NRS score, sleep quality scale, and I-FEED scores were not objective indicators; therefore, they may have affected the efficacy of the evaluation. Third, oxygenation was assessed using only arterial blood gas, and pulmonary function was not assessed. Also, prolonged pain intensity and chronic pain were not assessed. Fourth, the surgical method was not standardized. However, we considered this desirable because the different surgical procedures almost reflected real-world clinical practice situations. Fifth, we evaluated the effect of the TAPB only on the morning of the first postoperative day. A more exact operation time should be tested since the degree of pain may be affected by multiple circulating hormone levels that keep changing all day.

## Conclusions

In conclusion, rescue analgesia with the TAPB on the first postoperative day alleviated pain, enhanced oxygenation, and improved sleep quality following abdominal surgery; however, its effect on gastrointestinal function and long-term QoL requires further research.

## Data Availability

The datasets presented in this study can be found in online repositories. The names of the repository/repositories and accession number(s) can be found below: Data are available on Mendeley at: https://data.mendeley.com/datasets/nzvb7y39g6/1.
